# Description of an Apparatus Intended to Facilitate the Treatment of Fractures of the Lower Extremity

**Published:** 1833-10

**Authors:** 


*Description of an Apparatus intended to facilitate the Treatment of Fractures of the Lower Extremity.* By T. M. Greenhow, M.R.C.S. London, 1833. 8vo. pp. 22.

It would be hopeless to endeavour to give a description of Mr. Greenhow's apparatus, without giving a plate of it also, (which is of course quite out of the question.) But Mr. Greenhow has thought fit, in a somewhat novel

* Probably this should be Cetrach.—Editor.

fashion for a regular practitioner, to append to his pamphlet the favourable testimonials of a dozen eminent London surgeons. Sir Astley Cooper and Sir Charles Bell, with Messrs. Brodie, Guthrie, Mayo, Travers, &c., "have had much pleasure in examining," "have been much gratified in seeing," and so on, the apparatus. Now all this demonstrates, to our mind, that Mr. Greenhow's invention is of a good sober mediocrity. Were it very bad indeed, these excellent surgeons would not have yielded this handsome crop of laudatory notes: were it very good, Mr. Greenhow, who is an infirmary surgeon, would have known by experience that those who have to treat fractures, instead of praising it, would adopt it. It is surprising how little set panegyric is required by a really good invention, whether in or out of surgery: it asks no advertisements, no written testimonials,—*volitat vivu' per ora virûm.*

